# Starting CT-guided robotic interventional oncology at a UK centre

**DOI:** 10.1259/bjr.20220217

**Published:** 2022-03-21

**Authors:** Edward W Johnston, Jodie Basso, Jessica Winfield, James McCall, Nasir Khan, Christina Messiou, Dow-Mu Koh, Nicos Fotiadis

**Affiliations:** Royal Marsden Hospital, London, UK; Royal Marsden Hospital, London, UK; Royal Marsden Hospital, London, UK; Royal Marsden Hospital, London, UK; Royal Marsden Hospital, London, UK; Royal Marsden Hospital, London, UK; Royal Marsden Hospital, London, UK; Royal Marsden Hospital, London, UK

## Abstract

**Objective:**

A commercially available CT-guided robot offers enhanced abilities in planning, targeting, and confirming accurate needle placement. In this short communication, we describe our first UK experience of robotic interventional oncology procedures.

**Methods:**

We describe the device, discuss installation, operation, and report upon needle insertion success, accuracy (path deviation; PD and tip deviation; TD), number of adjustments, complications, and procedural success.

**Results:**

Nine patients (seven males), median age 66 years (range 43–79) were consented for biopsy or ablation between March and April 2021. Needle placement in biopsy was more accurate than ablation (median 1 *vs* 11 mm PD and 1 *vs* 20 mm TD) and required fewer adjustments (median 0 *vs* 5). No complications arose, and all procedures were successful (diagnostic material obtained or complete ablation at follow-up).

**Conclusion:**

Short procedure times and very high levels of accuracy were readily achieved with biopsy procedures, although tumour ablation was less accurate which likely reflects higher procedural complexity.

**Advances in knowledge:**

Achieving highly accurate robotic biopsy with is feasible within a very short time span. Further work is required to maximise the potential of robotic guidance in tumour ablation procedures, which is likely due to higher complexity giving a longer learning curve.

## Introduction

The objective in non-vascular, CT-guided interventional radiology (IR) is to accurately place a needle from a percutaneous entry point to a target via a safe path for diagnostic or therapeutic purposes. Conventional practice involves planning needle paths on axial CT images (in a single plane) with iterative ‘freehand’ manual needle targeting, where initial placement is usually unsatisfactory and multiple adjustments made. However, this approach can cause collateral tissue damage and increased complications, high radiation doses from sequential acquisitions and prolonged or distressing procedures,^1,2^ particularly where there is little room for error or complex approaches such as out-of-plane trajectories or multiple needles are required.

Devices which improve planning, targeting and confirmation could address these problems and potentially expand the horizons of non-vascular IR. A stereotactic CT-guided robot (Perfint MAXIO) is commercially available^3^ and offers enhanced capabilities in all of these functions. Initial studies have demonstrated more accurate needle placement,^4,5^ shorter procedure times^6^ and lower radiation dose^7^ than conventional freehand techniques, although the device has not been evaluated in the UK. The overall aim of this work is to report our initial experience in robotic guidance for interventional oncology applications. We describe the device, discuss installation, operation, and report our experience in a small cohort of patients undergoing biopsy and ablation procedures.

### MAXIO robot

The robot (MAXIO^™^; Perfint Healthcare, Chennai, India) has been described in detail previously.^4^ The device weighs 250 kg, measures 131.0 cm (height) x 77.5 cm (width) x 85.0 cm (depth) and has four swivel wheels which facilitate transfer to a 2 mm thin metallic floor mounted docking plate that calibrates the device to the scanner prior to use to achieve consistent stereotactic conditions.

**Figure 1.**
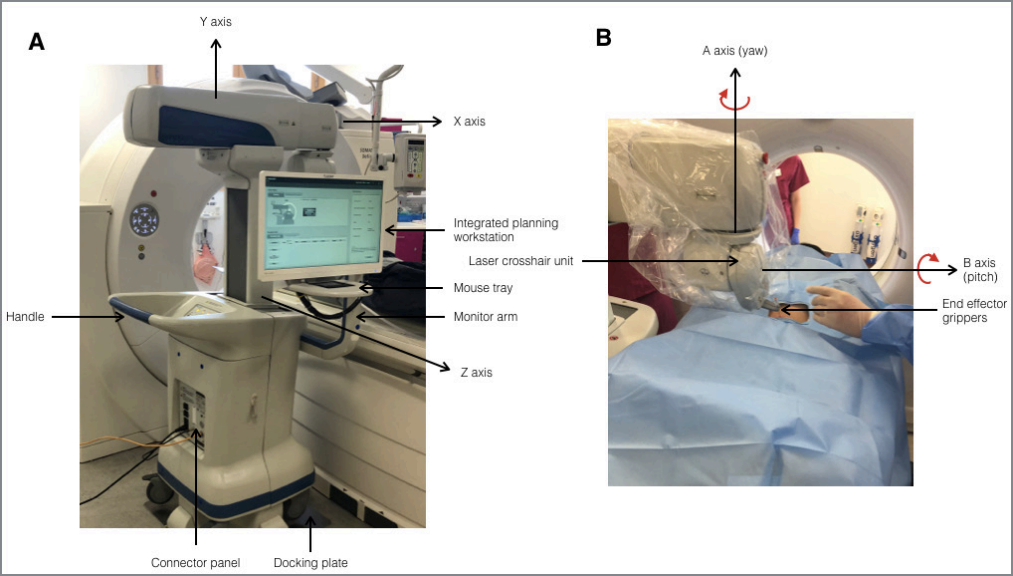
Annotated picture of the MAXIO robot (Perfint Healthcare, Chennai, India). (A) rear view (B) front view. Axes X, Y, Z, A and B are the five degrees of freedom of the electromechanical arm

An annotated picture of the device is shown in Figure 1.

DICOM data sets are sent from the scanner directly to the device via an ethernet cable in a near-instantaneous process. The integrated Windows operated planning workstation uses customised software on which up to six needle paths may be planned using multiplanar reformats for improved 3D appreciation.

After plan verification, a CT table position is stipulated by the workstation for first needle insertion to move the target to a suitable location the Z-direction. Each trajectory (based upon CT co-ordinates) is then translated to object space (*i.e.* stereotaxy) by an electromechanical arm. The arm has 5 degrees of freedom (linear axes X, Y, Z and angular axes A and B (pitch, rotation around the x-axis; and yaw, rotation around the y-axis)), reaching between −90° and+60° in orbital angulation and ±60° in craniocaudal angulation. The ‘end effector’ grippers, located at the end of the arm hold disposable plastic needle guides through which 22–11G needles are manually placed by an Interventional Radiologist (to the hub) to reach the target. The end effector grippers are released, and the robotic arm withdrawn, leaving the free needle *in situ*.

The patient is scanned again to assess needle position, and this second acquisition is sent to the device workstation to compare differences in planned *vs* actual needle paths using CT image fusion, again almost instantaneously. If needle position is unsatisfactory, it can be adjusted manually. The technique assumes the target does not move between the initial acquisition and needle insertion, which usually necessitates vacuum immobilisation ± respiratory motion control.

### Setup

Institutional permissions were gained and a DICOM network node established, with a static IP address and network port for image transfer. The device was delivered to the hospital, and a company engineer attended for installation which involved unpacking, fixing the docking plate semi-permanently to the scanner floor, establishing communication and calibrating with CT co-ordinates. The installation process took 3 days and was performed after working hours. A training session was held for participating radiographers and radiologists over a few hours and comprised CT acquisition, docking/undocking procedures, planning, targeting, and confirming needle placement using a simple training phantom supplied by the manufacturer. Company-supported clinical procedures then took place from the following day. Although usually supervised for a month, in our case supervised procedures were unexpectedly limited to a week due to the COVID-19 pandemic case surge in India, such that four procedures were supported on site, with further support available using video telementoring.

## Methods and materials

### Patients

Institutional permissions were gained, and data were collected as part of a service evaluation using this FDA approved, CE marked medical device. Written informed consent for procedures was obtained from all patients, as standard of care.

We performed robotic procedures on all patients referred for CT-guided biopsy of retroperitoneal or pelvic tumours or microwave ablation of liver tumours during the study period, due to a lack of respiratory excursion (natural in the former, and from anaesthesia in the latter).

### Procedures

Our team comprised (i) two Consultant Interventional Radiologists for planning, robot operation, needle insertion and image confirmation (EJ, 4 years’ experience in Interventional Radiology including >200 ablations and NF, 19 years’ experience including >800 ablations), (ii) two radiographers for image acquisition and CT table movement, (iii) two nurses for sedation, monitoring patient motion and equipment preparation. An anaesthetist and operating department practitioner were also part of ablation procedures. Patients were consented and a team brief was carried out. The device was switched on, docked and patients transferred to the scanner room where a World Health Organisation (WHO) safety checklist was completed.

#### Preparation

Conscious sedoanalgesia was given for biopsy procedures. For ablation procedures, patients were anaesthetised using full muscle paralysis and high frequency jet ventilation to control for respiratory motion and minimise target excursion. Full body immobilisation was performed in a suitable position with a vacuum mattress technique (Klarity Vacuum Bag, OH) and tightly applied CT table straps. The left lateral oblique position provided more access to the right posterior liver segments for ablation procedures.

A sterile field was created, preparing ‘wider’ than for conventional freehand procedures to avoid touching the patient after scanning (causing the tumour to move) combined with the uncertainty of needle entry.

#### Acquisition and planning

CT images were acquired in the axial plane (with or without intravenous contrast) using a 3 mm slice thickness and 1 mm slice interval. Planning was then carried out using the workstation.

#### Execution

Local anaesthetic was administered through the needle guide, a cut made in the skin and the co-axial needle or ablation antenna inserted. Biopsies were performed using 15*G* × 11.1 cm or 14.8 cm co-axial needles (Argon Medical, Frisco, TX), and ablations using 15 or 17*G* × 15 or 20 cm antennae (PR15 or PR20 (XT), NeuWave Medical, J&J, NJ).

#### Evaluation

**Figure 2.**
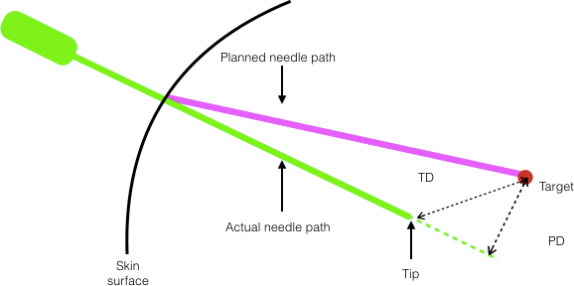
Schematic diagram of first placement needle accuracy metrics. PD, the shortest (perpendicular) distance from the needle path to the target, extending the needle path line to a hypothetical line beyond the actual tip position (if necessary). TD, the Euclidian distance between the actual and planned needle tips. PD, path deviation; TD, tip deviation

An unenhanced control CT was performed with needles *in situ* with the same acquisition parameters as the planning scan. Analysis of fused planning and control CT images enabled first placement path deviation (PD) and tip deviation (TD) to be measured using the system’s confirmation software (Figure 2). If needle position was satisfactory, the biopsy or ablation was performed. If unsatisfactory, the needle was adjusted manually, and checked again with repeat control CT/fusion software.

### Analysis

Reasons for unsuccessful robotic first needle insertion were documented. First placement PD, TD, number of manual needle adjustments, radiation dose (total milliamp seconds, mAs and dose–length product (DLP), mGy*cm), door-to-door procedure time, and duration of hospital stay were recorded. Complications were categorised according to the Society of Interventional Radiology (SIR) classification system.^8^ Successful biopsy procedures were defined as obtaining material satisfactory for histological diagnosis and successful ablation as complete coverage of the target tumour by the ablation zone on 6-week follow-up imaging.

## Results

Nine patients (seven males), median age 66 years (range 45–79) were consented for procedures between March and April 2021. No patients declined participation. A total of five biopsy (four retroperitoneal, one pelvic), and four microwave liver ablation procedures (for seven tumours) were attempted. Median target size was 26 mm (range 5–54). Baseline demographic and procedural information is provided in Table 1.

**Table 1.** Baseline patient demographic and procedural information

Patient number	Age	Sex	Organ	Procedure	Histology	Targets	Size (mm)	Planned needles	Liver segment	Supported
1	45	F	Adrenal	Biopsy	Lung adeno	1	54	1	-	Yes
2	66	M	Liver	Ablation	Adenoid cystic	2	26, 26	4	6	Yes
3	67	M	Adrenal	Biopsy	B-cell lymphoma	1	35	1	-	Yes
4	66	M	Presacral	Biopsy	Myelolipoma	1	44	1	-	Yes
5	48	M	Liver	Ablation	Rectal adeno	1	5	1	8	No
6	57	M	Adrenal	Biopsy	Small cell lung	1	35	1	-	No
7	79	M	RP	Biopsy	Haematoma	1	47	1	-	No
8	70	F	Liver	Ablation	Breast adeno	2	10, 14	2	5,7	No
9	43	M	Liver	Ablation	Colorectal adeno	2	12, 16	2	7	No

RP, retroperitoneal; adeno, adenocarcinoma.

### Biopsy procedures

**Figure 3.**
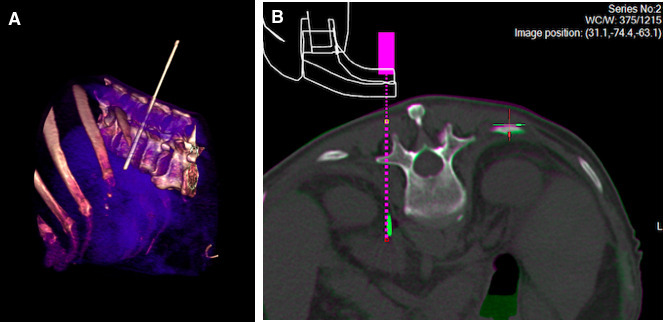
Example images from a successful robotic biopsy of a left adrenal metastasis in a 67-year-old man. (A) Volume rendered CT image demonstrating the out-of-plane approach taken by a rigid 15-gauge x 11.1 cm co-axial needle, as to avoid the lung base. (B) Screenshot from the MAXIO planning software showing fused unenhanced planning and control CT images. The dotted pink line demonstrates the planned needle path on the planning CT, and the solid green line is the distal aspect of the needle (for illustrative purposes, tip out of plane) on the control CT, with 1 mm path deviation and tip deviation.

Four of five biopsies had successful robotic first needle insertion, including two patients in whom negative samples were obtained at local hospitals. The unsuccessful robotic first needle insertion arose due to brisk patient motion at infiltration of local anaesthesia. PD was 1 mm in all four biopsy procedures and TD 1 mm in three of four biopsy procedures, and initially 9 mm in one biopsy procedure after initial placement, adjusted to 1 mm by manually advancing along the same needle path. Example images from a successful biopsy procedure are shown in Figure 3.

Robotic biopsy procedures had median radiation doses of 1483 total mAs (range 791–2597) and 295 mGy*cm DLP (range 147–558), and a median ‘door-to-door’ procedure time of 48 min (range 20–59).

### Ablation procedures

**Figure 4.**
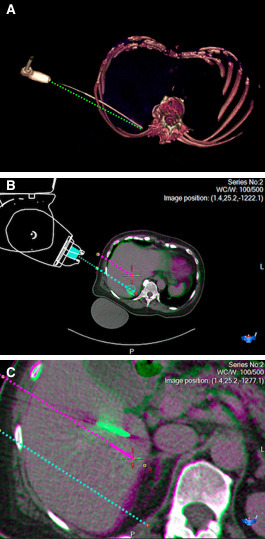
Example images from a microwave ablation procedure for two breast cancer liver metastases in a 70-year-old woman. (A) Volume rendered CT image showing the antenna path, and an annotated straight dotted green line to illustrate bending of the 17-gauge x 20 cm microwave ablation antenna. (B) Snapshot of fused CT images (before and after needle placement) from the MAXIO software showing accurate first antenna placement, with planned needle path (dotted blue line), *vs.* antenna tip (solid green) showing clinically acceptable path and tip deviation of 5mm each. (C) Fused CT images, showing less accurate antenna placement for the other lesion, with planned needle path (dotted pink line), *vs.* antenna tip (solid green) showing path and tip deviation of 16 mm and 20 mm respectively, necessitating manual repositioning.

Robotic first needle insertion was unsuccessful in the first two ablation procedures due to (i) patient motion during skin preparation (such that we moved this step to before the planning scan in subsequent procedures) and (ii) unintentional undocking of the robot due to human error. The subsequent two liver ablations (two target tumours each) were targeted with a single antenna per tumour. One antenna did not require adjustment (5 mm PD and TD) although the other three antenna placements required a median of five manual adjustments. Examples of antenna placement during ablation procedures are shown in Figure 4.

Robotic ablation procedures had median radiation doses of 6577 total mAs and 1399 mGy*cm DLP, median door-to-door procedure time 194 min. Complete ablation was seen in all tumours at 6-week follow-up.

All patients undergoing biopsy procedures were discharged the same day, and all ablation procedures after a 1-night stay without complications. Detailed information regarding procedures is provided in Table 2.

**Table 2.** Detailed information regarding procedures

Patient number	Procedure type	Changed	Reason	PD (mm)	TD (mm)	Adjustments	Time (mins)	mAs	DLP (mGycm)	Stay (days)
1	Biopsy	No	-	1	1	0	20	791	147	0
3	Biopsy	No	-	1	1	0	59	1168	236	0
*4*	*Biopsy*	*Yes*	*Patient motion*	*-*	*-*	*(6)*	*(25)*	(*2815*)	(*513*)	(*0*)
6	Biopsy	No	-	1	9	1	38	2597	558	0
7	Biopsy	No	-	1	1	0	58	1797	354	0
**Median**				**1**	**1**	**0**	**48**	**1483**	**295**	**0**
*2*	*Ablation*	*Yes*	*Patient motion*	*-*	*-*	(*0, US*)	(*228*)	(*2247*)	(*470*)	(*1*)
*5*	*Ablation*	*Yes*	*Robot undocking*	*-*	*-*	*(3)*	(*185*)	(*4248*)	(*889*)	*(1)*
8	Ablation	No	-	5, 16	5, 20	0, 7	198	6015	1299	1
9	Ablation	No	-	10, 12	20, 22	4, 6	189	7138	1498	1
**Median**				**11**	**20**	**5**	**194**	**6577**	**1399**	**1**

DLP, dose-length product; NB: Changed, changed to conventional freehand technique; PD, path deviation; TD, tip deviation; Time, total ‘door-to-door’ procedure time; US, ultrasound; mAS, total milliamp seconds.

Robotic procedures that were fully converted to freehand placement are in italics, with results in parentheses not contributing to calculation of medians.

## Discussion

We report our initial experience using a commercially available CT-guided interventional robot which offers enhanced abilities for planning, targeting and confirmation of needle placement. For biopsy procedures, we achieved median PD and TD of 1 mm with a single instance of needle adjustment, meaning the device shows potential for highly accurate biopsies with short procedure duration (acceptable for NHS use), after a single training session lasting a few hours.

Needle positioning in ablation procedures was less accurate (11 mm median PD, 20 mm TD) and required more adjustments (median 5) with longer duration and higher radiation doses, likely due to higher procedural complexity and longer learning curves. The largest study of liver ablation using the device (30 patients) reported mean TD of 5.8 mm and 1.1 readjustments,^5^ meaning higher levels of accuracy can achieved as experience builds. There are several ways in which ablation antennae differ from co-axial biopsy needles including diameter, length, flexural rigidity, tip sharpness and asymmetry, where needle bending was not observed in biopsy, unlike ablation procedures. Furthermore, the requirement of antennae to be connected to the ablation machine during positioning and scanning might result in less reliable placement and greater potential for migration. These issues might be addressed by initially placing co-axial needles, through which ablation antenna are inserted once appropriate position is confirmed,^1,9^ or by trying other antennae with different properties. It is noteworthy that needle placement accuracy in our biopsy cohort was higher than a phantom study using the same device, where mean TD was 6.5 mm,^4^ which could also be attributable to different needles or differing “tissue” properties.

Limitations of the device include diminished haptic feedback, inability to account for needle/tissue interactions, requirement for immobilisation and manual rather than robotic needle adjustment. Our small study was also performed in a single specialist centre with relatively narrow procedural scope, and a curtailed mentoring period due to the SARS-CoV-2 B.1.617 variant surge in India.^10^

To realise the benefits of robotic guidance whilst maintaining patient safety, the need for multidisciplinary collaboration with radiographers and nursing staff cannot be overstated. We also recommend selecting simple biopsy procedures before more complex tumour ablation procedures are undertaken. Further work will include expanding the technique to a larger cohort of patients where the learning curve will be examined and for precision biopsy with functional imaging modalities to target biologically deterministic components and (de)validate novel imaging biomarkers,^11^ as has been applied to PET/CT by another group using the same device.^12,13^ Ablation procedures might also benefit from image fusion for ablation zone confirmation^14^ and targeting CT/ultrasound occult tumours.^15^

## Conclusion

We report our initial experience with CT guided robotic interventions at a UK centre. Whereas short procedures times and very high levels of accuracy were observed with biopsy procedures, antenna placement in tumour ablation was less accurate which likely reflects higher procedural complexity.
